# Diversification of Fungal Chitinases and Their Functional Differentiation in *Histoplasma capsulatum*

**DOI:** 10.1093/molbev/msaa293

**Published:** 2020-11-13

**Authors:** Kristie D Goughenour, Janice Whalin, Jason C Slot, Chad A Rappleye

**Affiliations:** 1 Department of Microbiology, Ohio State University, Columbus, OH; 2 Department of Plant Pathology, Ohio State University, Columbus, OH

**Keywords:** chitinase, GH18 family, fungi, *Histoplasma*

## Abstract

Chitinases enzymatically hydrolyze chitin, a highly abundant and utilized polymer of *N*-acetyl-glucosamine. Fungi are a rich source of chitinases; however, the phylogenetic and functional diversity of fungal chitinases are not well understood. We surveyed fungal chitinases from 373 publicly available genomes, characterized domain architecture, and conducted phylogenetic analyses of the glycoside hydrolase (GH18) domain. This large-scale analysis does not support the previous division of fungal chitinases into three major clades (A, B, C) as chitinases previously assigned to the “C” clade are not resolved as distinct from the “A” clade. Fungal chitinase diversity was partly shaped by horizontal gene transfer, and at least one clade of bacterial origin occurs among chitinases previously assigned to the “B” clade. Furthermore, chitin-binding domains (including the LysM domain) do not define specific clades, but instead are found more broadly across clades of chitinases. To gain insight into biological function diversity, we characterized all eight chitinases (Cts) from the thermally dimorphic fungus, *Histoplasma capsulatum:* six A clade, one B clade, and one formerly classified C clade chitinases. Expression analyses showed variable induction of chitinase genes in the presence of chitin but preferential expression of *CTS3* in the mycelial stage. Activity assays demonstrated that Cts1 (B-I), Cts2 (A-V), Cts3 (A-V), Cts4 (A-V) have endochitinase activities with varying degrees of chitobiosidase function. Cts6 (C-I) has activity consistent with *N*-acetyl-glucosaminidase exochitinase function and Cts8 (A-II) has chitobiase activity. These results suggest chitinase activity is variable even within subclades and that predictions of functionality require more sophisticated models.

## Introduction

Chitin is a (1,4)-β-linked *N*-acetyl-d-glucosamine (GlcNAc) polymer. As the second most abundant biopolymer after cellulose (Tharanathan and Kittur 2003), chitin and its deacetylated derivative chitosan are abundant sources of organic carbon and nitrogen that have many potential industrial uses, from biomedical to agricultural to water engineering (Ravi Kumar 2000; Zargar et al. 2015). Consequently, there is a great interest in identifying enzymes that efficiently hydrolyze chitin into more soluble mono- and oligomers of GlcNAc. Chitin degrading enzymes also have potential applications in the breakdown of the chitinous structures of fungal and arthropod agricultural pests (Hamid et al. 2013).

Chitinases (E.C 3.2.1.14) are glycosyl hydrolases that are found in a wide range of plants, bacteria, and fungi. For plants and bacteria, which lack chitin, chitinases play roles primarily in defense against fungi and/or arthropods. Chitin is an important structural component of the fungal cell wall, ranging from 0.5% to 5% in yeasts to ≥20% in some filamentous fungi (Hartl et al. 2012). Yeast-form fungi possess relatively few chitinases (e.g., two in *Saccharomyces cerevisiae*) compared with the 10–20 chitinases encoded in some filamentous fungal genomes (Seidl 2008). The expansion of chitinases in filamentous fungi has prompted investigation of chitinase roles in formation of hyphal structures (Seidl 2008). Although some studies have shown select chitinases are specifically induced during hyphal formation (Takaya et al. 1998; Gruber, Kubicek, et al. 2011), many other chitinases are not (Duo-Chuan 2006; Seidl 2008; Gruber, Vaaje-Kolstad, et al. 2011) suggesting alternative roles for diverse chitinases. For example, there is evidence that specific chitinases facilitate mycoparasitism of other fungi by *Trichoderma* (Cruz et al. 1992; Seidl et al. 2005; Boer et al. 2007).

Fungal chitinases are characterized by the presence of the gycoside hydrolase 18 family (GH18) (Seidl 2008) domain. Additional motifs often found in chitinases include: an N-terminal signal peptide that serves to direct secretion, a serine/theronine-rich region, one or more chitin-binding domains (CBDs), or LysM domains. The LysM domain enables binding to polysaccharides such as peptidoglycan and chitin (i.e., a CBD). However, none of these motifs beyond the GH18 domain is conserved across all fungal chitinases or required for a protein to be considered a chitinase (Duo-Chuan 2006).

A three-clade classification system for fungal chitinases (clades A, B, and C) emerged from several investigations into the diversity of GH18-domain-containing enzymes among fungi (Seidl et al. 2005; Karlsson and Stenlid 2008, 2009; Seidl 2008). Although some studies have further divided the B clade into D and E clades, this appears to be a result of their specific data sets rather than a widely applicable feature (Junges et al. 2014). Clade A chitinases are between 40 and 50 kDa and were previously reported to have no CBD. These are the most well studied of the three clades (Seidl 2008). Clade B chitinases are variable in size and also in the presence and number of CBDs. Clade C chitinases are distinguished by a significantly larger size (140–170 kDa) due to extension at the C-terminus. Clade C chitinases were also proposed to be defined by the presence of multiple CBDs, and especially LysM motifs (Seidl et al. 2005; Karlsson and Stenlid 2008, 2009). Clade C chitinases are the least well characterized of all the clades; few members have been included in previous phylogenetic studies, and none have been functionally characterized (Duo-Chuan 2006; Seidl 2008). However, this three clade classification of fungal chitinases is based on a limited number of fungal genomes, and it remains to be determined if it is robust to more in-depth taxon sampling (Seidl et al. 2005; Karlsson and Stenlid 2008, 2009).

Although many fungal chitinases have been transcriptionally profiled, the characterization of fungal chitinase enzymatic specificity is particularly limited, leaving broad assumptions about clade-specific functions untested. For example, Clades A and C are class V chitinases, which are generally assumed to be exochitinases based on modeling of the conserved binding groove as deep and tunnel shaped (Duo-Chuan 2006; Seidl 2008; Hartl et al. 2012). Clade B corresponds to class III chitinases and is predicted to be endochitinases due to the modeling of their binding grooves as shallow and open (Duo-Chuan 2006; Seidl 2008; Hartl et al. 2012). Although some of these assumptions are supported by the activities of specific chitinases (i.e., the CHIT33 and CHIT42 chitinases of *T. harzianum*; Boer et al. 2007; Lienemann et al. 2009), these chitinases have multiple activities and further examples need to be studied to establish clade-defining characteristics (Hartl et al. 2012). The inconsistent use of diverse chitin substrates (e.g., crustacean chitin or fungal chitin) further confounds the reliability of assumptions about functional diversity within and among clades. Although there are reports of complete chitinase or clade/subclade-specific chitinase transcriptional or deletion studies (Dünkler et al. 2005; Alcazar-Fuoli et al. 2011; Gruber, Kubicek, et al. 2011; Gruber, Vaaje-Kolstad, et al. 2011), enzymatic studies have not been as comprehensive.

In this study, we identified the GH18 domain proteins encoded in 373 published fungal genomes to conduct a more complete analysis of their distribution and evolution. In addition, we provide initial expression analysis and enzymatic characterization of the chitinase enzymes produced by the thermally dimorphic fungus, *Histoplasma capsulatum.* This organism has distinct and tightly controlled morphologies (mycelia or yeasts), each of which has a specific lifestyle (environmental saprobe or pathogen of mammals, respectively) potentially allowing for identification of specific roles for different chitinases. These chitinases provide new enzymatic examples from each of the major clades, including the enzymatic activity of the first characterized clade C chitinase.

## Results

### Diversification of Fungal GH18 Domains

We identified 3,888 GH18 domain-containing proteins in 373 publicly available fungal genomes (supplementary data 1, Supplementary Material online) using a hidden Markov model (HMM) search (Eddy 2009). About 494 of these proteins contain the LysM CBD (supplementary data 2, Supplementary Material online) considered to be characteristic of the C clade chitinases in fungi (Seidl et al. 2005; Seidl 2008). About 1,250 CBDs (i.e., ChtBD1, ChtBD3, COG3979, CBM_1, and CBM_19) were also identified in the GH18 domain-containing proteins (Marchler-Bauer et al. 2017).

**Fig. 1.**
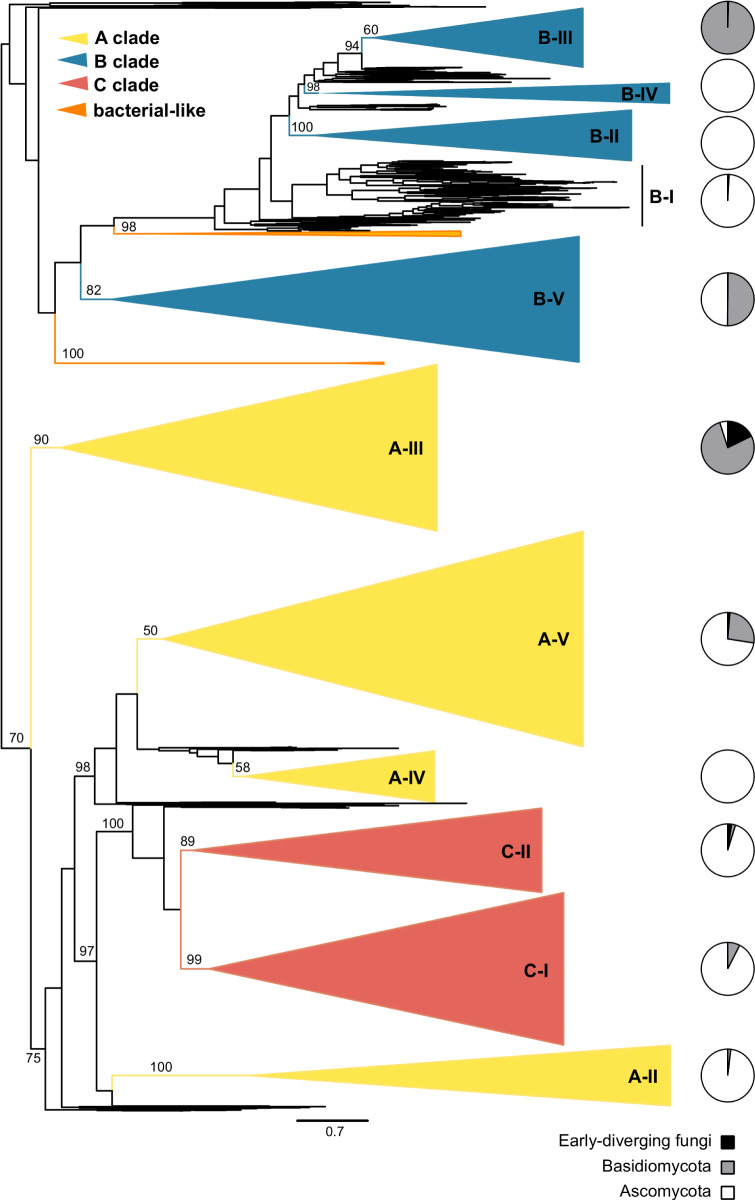
Phylogenetic analysis of fungal GH18 domains. Phylogenetic tree depicts relationships among 3,888 fungal chitinase proteins based on alignment of their glycosyl hydrolase (GH18) domains. Trees were built using maximum-likelihood methods (IQ-Tree) and branch support assessed by 1,000 ultrafast bootstrap replicates (values indicated by numbers at the root of common ancestor branches). Major groups corresponding to the previously named A, B, and C clades are indicated by color (yellow, blue, red, respectively). Clades comprised bacterial-like GH18 proteins are indicated (orange). Collapsed subclades, indicated as triangles with names to the right, are defined by the most recent common ancestor of previously categorized chitinases, in some cases extended to supported clades consistent with the fungal phylogeny. The tree is rooted along the midpoint, which generates the B versus AC clade split that has been widely accepted in the literature (Seidl et al. 2005; Karlsson and Stenlid 2008, 2009). Pie charts at the far right show the fraction of the constituent chitinase proteins in each clade occurring in early-diverging fungi (black), Basidiomycota (gray), and Ascomycota (white).

Alignment of the GH18 domains of these proteins was 1,840 characters; however, the C-terminus of the alignment is not well-conserved and occasionally absent particularly among members of the B clade ( including functionally validated chitinases; Dünkler et al. 2005; Hurtado-Guerrero and van Aalten 2007 ). Maximum likelihood phylogenetic analysis using IQ-Tree was used to establish evolutionary relationships among the fungal chitinases and to infer support for clades and subclades defined by the common ancestor of previously classified chitinases, with some slight expansion for closely related but previously unstudied taxa (fig. 1, table 1, and supplementary data 3, Supplementary Material online). This identified the previously reported A + C clade, although this was not well-supported (70% of rapid bootstraps [RB]). The B clade was not supported. Constituent subclades, determined by identifying the most recent common ancestor of previously categorized chitinase subclades (Seidl et al. 2005; Karlsson and Stenlid 2008, 2009), were generally recovered with variable support. Further expansion of subclades recovers more strongly supported nodes without encroaching on other subclades (fig. 1).

**Table 1.** Bootstrap Support Values for Subclades by Various Phylogenetic Analyses.

Subclade	GH18 IQ Tree	B or AC Clade IQ Tree	B or AC Clade RAxML
B-I	NA	98	10
B-II	100	100	75
B-III	60	69	17
B-IV	98	84	17
B-V	82	97	54
A-II	100	100	100
A-III	90	100	68
A-IV	58	67	75
AV	50	NA	11
C-I	99	91	68
C-II	89	80	36

**Fig. 2.**
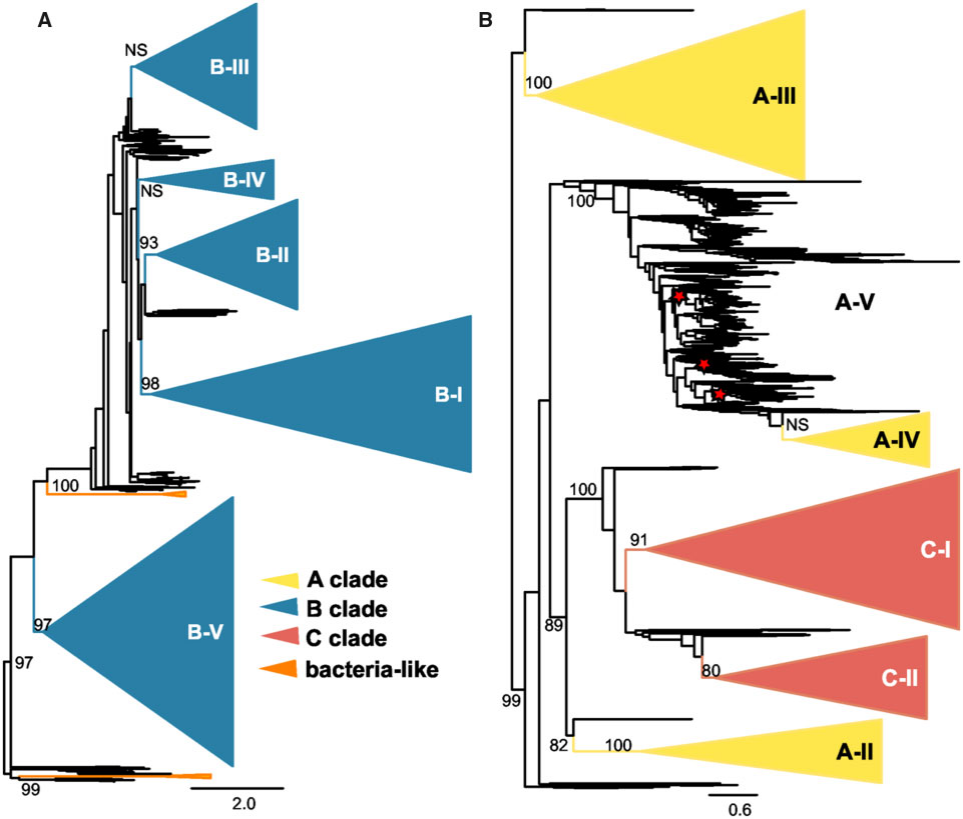
Phylogenetic analysis of fungal B and AC clades. Relationships among constituent proteins in the B and AC clades when analyzed as separate groups. Trees were built using maximum-likelihood methods (IQ-Tree) and branch support assessed by 1,000 ultrafast bootstrap replicates (values indicated as percentages at the base of the clade). Trees are rooted as in the complete GH18 IQ-Tree in figure 1. Collapsed subclades, indicated as triangles with names to the right, are defined by the most recent common ancestor of previously categorized chitinases, in some cases extended to supported clades consistent with the fungal phylogeny. (*A*) IQ-Tree-based phylogeny of B clade chitinase proteins with subclades indicated and collapsed (blue). (*B*) IQ-Tree-based phylogeny of chitinases belonging to the A (yellow) and C clades (red) showing the evolution of the C clade as a branch of the A clade. As subclade A-V is polyphyletic in the AC tree, the ancestral nodes giving rise to previously identified chitinases are indicated with red stars.

In order to retain more alignable characters and thereby reduce error introduced by homoplasy for finer resolution within clades and subclades, we separately analyzed sequences on either half of a bifurcation between the A + C clades and an assemblage containing all the B subclades in the complete tree (fig. 2). In the complete GH18 tree (fig. 1), we did not recover a B-I subclade and only the B-II and B-IV subclades received strong RB support (table 1). However, in the IQ-tree focused on B subclades, B-I (98% RB), B-II (100% RB), and B-V (97% RB) clades were supported, whereas clades B-III and B-IV were not supported with the current strict definition of subclades, and a B clade remained unsupported (fig. 2*A* and table 1). However, slight expansions of the subclades identified supported nodes consistent with the previous analyses. A RAxML analysis of this alignment also recovered these B subclades but without RB support, suggesting the finer scale topology is not always robust to differences in methodology (table 1 and supplementary data 3, Supplementary Material online).

For the branch in the entire GH18 tree containing the A and C clades (hereafter referred to as the “AC” clade), we recovered distinct, yet unsupported A-IV (58% RB) and A-V (50% RB) subclades after reclassifying one *T. reesei* gene (Chi18-5) from an A-V to an A-IV (fig. 1 and table 1). In the AC-only IQ-Tree (fig. 2*B*), the A-V subclade was not monophyletic. The A-II (100% RB, IQ-Tree) and A-III (100% RB, IQ-Tree) subclades were supported in all analyses (fig. 1 and 2*B*; table 1). The A-IV and A-V subclades were separated (98% RB, IQ-Tree) from the A-II and C clades (fig. 1). Although the C group is monophyletic within the AC clade (fig. 1), separation into subclade C-I was supported (99% RB), whereas subclade C-II was not (89% RB) in the complete tree (fig. 1, table 1, and supplementary data 3, Supplementary Material online). In the separate AC tree, C-II is not supported but would be supported by including sister sequences (fig. 2*B*). Interestingly, in our analysis clade C (both C-I and C-II) groups closely, with strong support, with A-II (97% RB in the all GH-18 IQ-Tree) to the exclusion of other A subclades (fig. 1 and supplementary data 3, Supplementary Material online). This is also characteristic of an unrooted phylogenetic tree (supplementary fig. S1, Supplementary Material online).

### Taxonomic Distribution of GH18 Chitinases

**Fig. 3.**
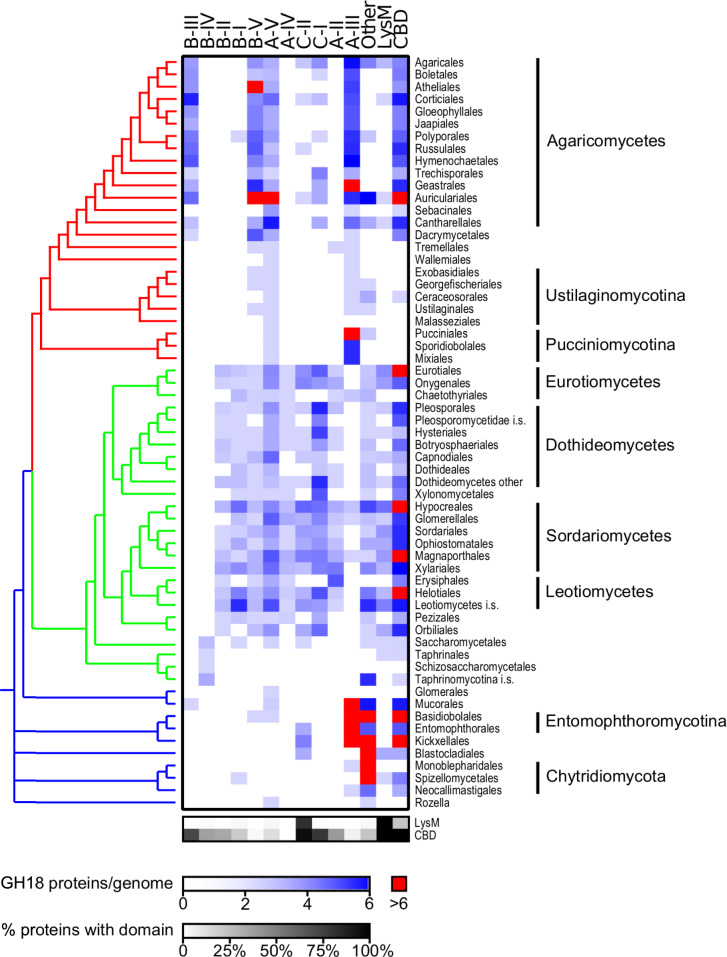
Distribution of chitinases among fungal taxa. Heat map showing the distribution of chitinase subclades across fungal orders. Shading indicates the average number of GH18 domain-containing proteins belonging to each subclade (columns) per genome with orders with highly expanded numbers (at least six per genome) of subclade chitinases indicated (red). Taxonomic relationships among fungal orders is presented for reference (left side) and colored for early-diverging fungi (blue), Ascomycota (green), and Basidiomycota (red). The right two columns of the heat map indicate the number of chitinases per genome with LysM or CBD domains, respectively. Lower gray-scale heat map indicates the percentage of the subclade proteins with LysM and CBD domains.

Each subclade hosts a distinct assortment of fungal taxa. The Ascomycota, particularly Leotiomycetes, Sordariomycetes, Eurotiomycetes, and Dothideomycetes, have the largest number of chitinases and highest diversity of chitinase subclades (fig. 3 and supplementary data 4, Supplementary Material online). B subclades-I, -II, and -IV are composed almost entirely of Ascomycota. The individual orders Malasseziales, Glomerales, Monoblepharidales, Neocallimastigales, and Rozella have the fewest chitinases per genome (e.g., 1 or 0.5 [Rozella]) and Auriculariales, Basidiobolales, Geastrales, and Xylariales have the most (greater than 17). The B-IV subclade consists entirely of Saccharomycetales, whereas the B-I and B-II subclades are composed of non-Saccharomycetales, Ascomycota. B-I consists of Leotiomycetes, Agaricomycetes, Dothideomycetes, Eurotiomycetes, Leotiomycetes, Sordariomycetes, and Xylonomycetes, with multiple orders of Dothideomycetes, Eurotiomycetes, and Sordariomycetes represented. B-II contains multiple orders of Eurotiomycetes, Dothideomycetes, Leotiomycetes, and Sordariomycetes. Subclade B-V contains a large number of Basidiomycota; however, several species of Ascomycota and a single early-diverging fungus (*Basidiobolus meristosporus*) are also represented. B-III is composed almost entirely of Basidiomycota with the exception of sequences from the early-diverging Mucorales.

Within the AC clade, subclades A-II and A-IV are composed mostly of Ascomycota with isolated Basidiomycota (Tremellales in A-II and Pucciniales in A-IV) (fig. 3). The A-II subclade consists of Eurotiomycetes, Sordariomycetes, Leotiomycetes, and Dothideomycetes, although within the Dothideomycetes the order Capnodiales and an unnamed order in Pleosporomycetidae were not represented. Only the Dothideales, Orbiliales, and Lecanorales were not represented in A-IV. A-III is composed of both Basidiomycota and early-diverging fungal chitinases with a few Ascomycota (Sordariomycetes). Malasseziales was the only order of Basidiomycota with no A-III chitinases, whereas in the early-diverging fungi Glomerales, Blastocladiales, and Rozella also lacked these chitinases. The A-V subclade contains Ascomycota, Basidiomycota, and early-diverging fungi. All Ascomycota and Basidiomycota were represented in this clade except one member of an unnamed class in Taphrinomycotina, and among early-diverging fungi, they are only found in Rozella, Mucorales, Glomerales, and Basidiobolales. The C-I subclade mostly contains Ascomycota with a small assortment of sequences from six orders of Agaricomycetes, whereas the C-II subclade contains both Ascomycota and a few early-diverging fungi (Kickxellales, Entomophthorales, and Blastocladiales). Relatively few chitinases are found in Botryosphaeriales.

### Patterns of GH18 Chitinase Diversification

Much of the GH18 chitinase phylogeny is consistent with vertical inheritance. More derived branches of the chitinase phylogeny frequently track the species phylogeny, particularly in the A subclades (A-V, A-IV, A-III, and A-II) and the B-V subclade. In keeping with the fungal phylogeny, the B-V subclade contains distinct Agaricomycetes, Eurotiomycetes, and Dothideomycetes groups, and a single species of Leotiomycetes within a Sordariomycetes group. Similarly, the A-IV subclade contains distinct clades of Sordariomycetes, Eurotiomycetes, Leotiomycetes, and Dothideomycetes. The A-II subclade has large separate Sordariomycetes and Dothideomycetes groups. The A-V subclade contains a distinct Saccharomycetes clade, but there are also multiple Agaricomycetes, Sordariomycetes, and Eurotiomycetes clades. There are large-scale divergences from the fungal tree, including a clade of Sordariomycetes within a clade otherwise composed of Agaricomycetes in the A-III subclade. Despite such grouping of related species, the branching order within GH18 phylogenies is overall complex, suggesting domain and gene duplications are common. Some subclades in particular (e.g., the C subclades) show few taxon-specific clades (fig. 3).

Part of the contrast with the species tree comes from specific supported instances of horizontal gene transfer (HGT) (supplementary fig. S2, Supplementary Material online). For example, two clades of mainly Hypocreales (Pezizomycotina) fungi were initially poorly-placed among the B subclades in the GH18 phylogenies. A reanalysis including fungal and nonfungal sequences at NCBI more confidently placed these sequences among bacterial chitinases (fig. 1), suggesting the clades originated through HGT, further reducing support for a B clade. One of these chitinases appears to have been acquired specifically by insect-pathogenic Hypocreales fungi (e.g., XP_006673222.1 *Cordyceps militaris*) from bacteria of an unknown lineage (supplementary fig. S2*A*, Supplementary Material online). This *C. militaris* chitinase is an endo-*N*-acetylhexosaminidase active on fucose-containing N-glycans (Huang et al. 2018). The other chitinase, found in a much larger group of fungi in Hypocreales and additional fungi in Pezizomycotina is supported to have been transferred from Actinobacteria to Hypocreales (e.g., XP_006670951.1 *C. militaris*, supplementary fig. S2*B*, Supplementary Material online) and later to other fungi. This single-domain chitinase is most similar to chitinase D (cd02871), which has hydrolytic and transglycosylation activity in bacteria (Vaikuntapu et al. 2016). Two other HGTs of chitinase D were supported (supplementary fig. S2*C*, Supplementary Material online), one from Streptomyces (Actinobacteria) to Arthrodermataceae (Eurotiomycetes), and the other from Micromonadaceae (Actinobacteria) to Auriculariales (Agaricomycetes). (Vaikuntapu et al. 2016). Still another group of insect-associated Hypocreales chitinases (e.g., KOM21536.1 *Ophiocordyceps unilateralis*, supplementary fig. S2*D*, Supplementary Material online) is dispersed in a clade of fungal chitinases otherwise composed of multiple paralogs from the arthropod-associated Kickxellomycotina. These chitinases are part of the expanded A-III subclade, and contain a GH18 domain and a signal secretion peptide. Outside Hypocreales, the amphibian gut symbiont *B. meristosporus* appears to have acquired a B-V chitinase with only a GH18 domain (Basme2_176417, supplementary fig. S2*E*, Supplementary Material online) from Agaricomycetes, possibly in Auriculariales, but a specific donor is not supported. Finally, another interphylum transfer appears to have occurred from Pezizomycotina to Panaeolus (Pancy2_12872, Agaricales, supplementary fig. S2*F*, Supplementary Material online). The nearest sequence, in *Uncinocarpus reesei* (Uncre1_6593), associates this last event with the dung decay niche. This is a C-II chitinase with two LysM and one CBD domain. Analyses comparing topological constraints were able to reject monophyly of the hypothetical donor groups for most, but not all HGTs, due to the complexity of the overall phylogeny and an absence of a reliable outgroup in some cases (supplementary data 5, Supplementary Material online).

### Evolution of Domain Architecture

Chitinases with multiple GH18 domains were rare, observed in only 11 cases. Of these, all contained two GH18 domains except *Psilocybe cyanescens* [Psicy2_12260], which contained three. All multi-GH18 domain proteins were in Basidiomycota; most were in Agaricales (9 of 11 cases) and there was one each in Polyporales and Pucciniales. Multi-GH18 domain proteins are not constrained to a specific subclade, and are found in the A-III (six cases), B-III (three cases), and B-V (two cases) subclades. Interestingly only five GH18 domains in multi-GH18-containing proteins, all in B subclades, were predicted to be active. In addition, both GH18 domains in multidomain A-III chitinases lacked conserved active site residues.

LysM domains occur in four chitinase subclades. Most are restricted to the C-II subclade (fig. 3 and supplementary data 2, Supplementary Material online), which also includes the previously described LysM-containing class III Hce2 (Homologs of *C. fulvum* Ecp2) effector proteins (Stergiopoulos et al. 2012). Some LysM domain-containing proteins formed a monophyletic group within the A-V subclade, and their LysM domain is similar to bacterial spore assembly proteins. Almost all other remaining LysM-containing chitinases, which include the C-I, one A-V member (Talma12_7383), and a B-I (Spoth2_113450) protein, share a different recent LysM common ancestor. There is evidence of additional LysM duplications of various ages that have resulted in phylogenetic diversity among LysM domains in the same protein (supplementary data 2, Supplementary Material online); however, the amino acid alignment is lacking sufficient characters to infer robust relationships among most LysM domains.

CBDs are dispersed across chitinases in early-diverging fungi, Ascomycota and Basidiomycota (fig. 3 and supplementary data 2, Supplementary Material online). Zoopagomycota (particularly Basidiobolomycetes) have comparatively large numbers of CBDs, and other early-diverging fungi (Mucoromycotina and Kickxellomycotina) have somewhat fewer. CBDs are widely dispersed through the classes and orders of Ascomycota, but in Basidiomycota, they are mostly restricted to Agaricomycetes (fig. 3). The earliest diverging B subclade, B-V, has a low percentage of chitinases with CBDs, whereas there are more of these domains in other subclades, particularly the B-III clade (fig. 3). In the AC super clade, most CBDs are found in the C-I and C-II subclades as well as in a low percentage of A-V class proteins (fig. 3).

### *Histoplasma capsulatum* Chitinases

#### Diversification and Taxonomic Distribution

**Fig. 4.**
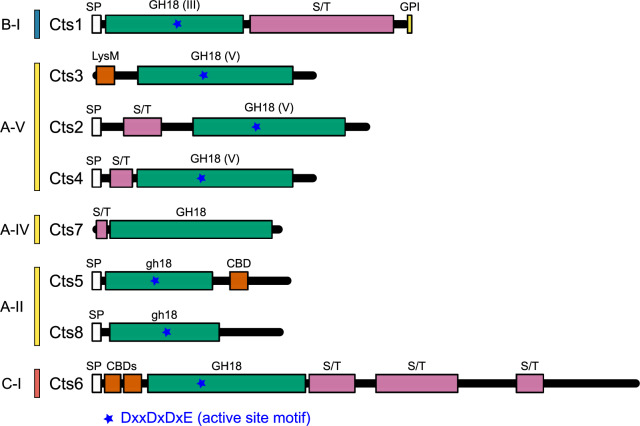
Domain and motif structure of *Histoplasma capsulatum* chitinases. The eight chitinase proteins (Cts) encoded in the *H. capsulatum* genome are schematically shown and grouped by subclade. Glycosyl hydrolase family 18 (GH18) domains (green) are indicated and noted as class III (GH18(III), class V (GH18(V)), or those with shortened length (gh18). Recognized chitin-binding domains (CBD and LysM) are indicated (orange). N-terminal signal peptides (SP; white) directing secretion of the protein, serine/threonine-rich regions (S/T; pink) as potential sites for O-linked glycosylation, and the motif for glycosylphosphatidylinositol attachment (GPI, yellow) are indicated. Presence and location of residues for the conserved DxxDxDxE motif for chitin hydrolysis are also shown (blue stars). Subclade to which each protein belongs is presented along the left.

The *H. capsulatum* genome encodes eight chitinase (Cts) enzymes, which are widely distributed in the GH18 phylogeny (fig. 4 and supplementary data 6, Supplementary Material online). Six genomes from diverse *H. capsulatum* strains were used for analysis (*H. capsulatum* G186AR, *H. capsulatum* G217B, *H. capsulatum H143*, *H. capsulatum* H88, *H. capsulatum* NAM1, and *H. capsulatum* TMU). Although the genomes generally contain the same chitinases, there are slight differences in clade distribution*. Histoplasma capsulatum* H143 and H88 (two representatives of the African strains) do not contain a C-I chitinase, whereas G217B has two that are 100% identical (likely the result of an assembly error). *Histoplasma capsulatum* TMU lacks the otherwise conserved A-IV chitinase, whereas *H. capsulatum* H143 is also missing the B-I chitinase. We cannot at this time rule out the absence of individual chitinases being due to errors in genome assembly (particularly with the low coverage of H143 at only 3-fold).

Cts1 is the only *H. capsulatum* sequence in the B clade. Cts6 is the sole C subclade protein (C-I) and also has the larger size characteristic of this subclade. The remaining *H. capsulatum* chitinases (Cts2, Cts3, Cts4, Cts5, Cts7, and Cts8) are all members of clade A (fig. 4). Cts2, Cts3, and Cts4 are members of the A-V subclade, Cts5 and Cts8 both belong to subclade A-II, and Cts7 is a member of subclade A-IV. Cts4 appears to be a very recent duplication of Cts2 as the closely related fungus *Blastomyces dermatitidis* has only a single Cts protein that is orthologous to both *H. capsulatum*’s Cts2 and Cts4 proteins. *Histoplasma* is the only genus in the eight Onygenales genera surveyed that contains chitinases in the A-II subclade, whereas other Onygenales species tend to contain members of the B-II subclade, which *Histoplasma* lacks. There are no A-III chitinases in *H. capsulatum*, consistent with other Onygenales surveyed (fig. 3).

*Histoplasma capsulatum* has only a single C-clade chitinase enzyme (C-I) even though the C clade (C-I and C–II) is expanded in multiple other Onygenales members. Among Eurotiomycetes, Onygenales and Eurotiales have similar distributions of chitinases among the subclades except as noted above. Chaetothyriales differ in that they contain A-III subclade chitinases but lack the B-II and C clade chitinases found in the other Eurotiomycetes (fig. 3).

#### Domain Architecture and Finer Motifs

The architectural diversity of *H. capsulatum* chitinases is consistent with the variability observed across the fungal kingdom. *Histoplasma capsulatum* chitinases look similar to those of other Onygenales. For example, other Onygenales have Cts3-like proteins with matching architecture (i.e., containing a LysM domain but no signal peptide). Most chitinases only have one to two CBDs even in C clade proteins. *Microsporum canis* has expanded numbers of CBDs and LysM domains corresponding to their expanded C clade, which is unusual for the Onygenales. At the individual protein level, domain architecture is variable among the A clade chitinases. Cts2, Cts3, and Cts4 are all in the A-V subclade. Of these, Cts2 and Cts4 have similar domain architecture consistent with a recent duplication, with secretion signals at the N-terminus and serine/threonine rich regions (fig. 4), which are common sites for O-linked glycosylation (Loibl and Strahl 2013). Cts3 contrasts with Cts2 and Cts4 by lacking the secretion signal and containing the only LysM domain among *H. capsulatum* chitinases. In subclade A-IV, Cts7 lacks a secretion signal and contains a serine/threonine rich region. Cts7 is also the only *H. capsulatum* chitinase that lacks conserved aspartate residues predicted to comprise the active site (Hartl et al. 2012). In subclade A-II, Cts5 and Cts8 both contain a secretion signal, but only Cts5 contains a recognizable CBD. The C-1 subclade chitinase, Cts6, possesses the N-terminal secretion signal, serine/threonine rich regions, and two CBDs. The B clade chitinase in *H. capsulatum*, Cts1 (B-I subclade), has a GPI attachment site in addition to a secretion signal suggesting this enzyme anchored to the cell surface of *H. capsulatum* cells. It also contains an extended serine/threonine rich region common among extracellular proteins. Thus, most *H. capsulatum* chitinases are predicted to be secreted enzymes and active on chitin substrates.

#### Expression of H. capsulatum Chitinases

**Fig. 5.**
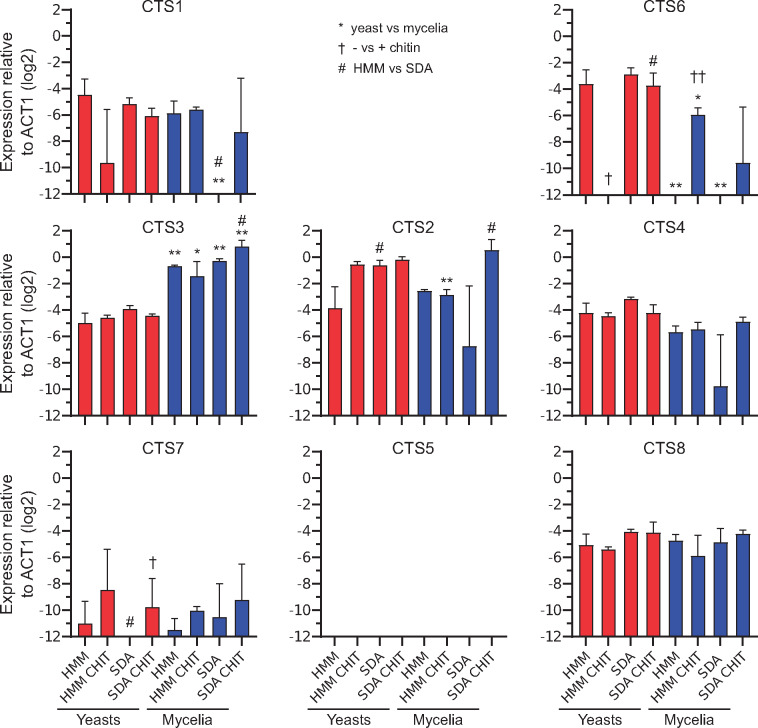
Expression of chitinase-encoding genes by *Histoplasma capsulatum* yeasts and mycelia. Expression of each chitinase-encoding gene (*CTS*) by *H. capsulatum* cells was measured using qRT-PCR. Transcription was determined for *H. capsulatum* cells grown on two media based either on basal cell culture medium (HMM) or based on glucose and peptone (Sauboraud’s dextrose; SDA). For some tests, media was supplemented with chitin (CHIT). Yeast (red bars) and mycelial (blue bars) phases were maintained by growth at 37 or 25 °C, respectively. Data show expression of each *CTS* gene relative to the expression of actin (*ACT*) and organized by subclades (B, cyan; A, magenta; C, green). Data represent the average ± SD among biological replicates (*n* = 3). Significant differences in expression between yeasts and mycelia (*), absence and presence of chitin (†), or HMM compared with SDA (#) are indicated for corresponding conditions differing by the single parameter. Single symbols indicate *P* < 0.05 and double symbols indicate *P* < 0.01 as determined by pairwise Student’s *t*-tests.

To provide insight into the physiological roles of diverse chitinases in *H. capsulatum*, the expression of each was determined under different environmental and nutritional conditions. As a thermally controlled dimorphic fungus, *H. capsulatum* provides an opportunity to ascribe morphology-specific (yeast- or mycelial-) roles to individual chitinases. Surprisingly, most chitinase-encoding genes (*CTS*) were expressed at constant, but low levels across most conditions including in the presence of exogenous chitin (fig. 5). Only *CTS3* (one of the A-V chitinases) was specifically upregulated in *H. capsulatum* filamentous cells (on average roughly 11-fold higher in mycelia compared with yeasts; fig. 5) suggestive of mycelia-specific functions. *CTS2* (encoding another A-V-class chitinase) was expressed at higher levels overall suggesting that the Cts2 protein could be important for general growth or the major functional chitinase under the tested conditions. *CTS4* (A-V) and *CTS8* (A-II) were expressed at low but consistent levels. *CTS1* (the only B-I chitinase) showed variable expression. *CTS6* (the C-I chitinase) also showed highly variable levels of expression that somewhat mirrored that of *CTS1* (fig. 5). *CTS7* (the only A-IV chitinase), which lacks the active site D-X-X-D-X-D-X-E residues (fig. 4), showed very low levels of expression. *CTS5* (A-II) expression was not detectable under any condition tested. Interestingly, the presence of exogenous chitin in the media did not consistently induce chitinase gene expression, with the exception of *CTS6* which was induced in the presence of chitin in most conditions except yeast-phase growth on rich media (fig. 5). Regardless, neither *H. capsulatum* yeast nor mycelia were able to grow on chitin as the carbon source of the growth media (data not shown). Thus, with the exception of *CTS3* and *CTS6*, expression studies did not reveal specific environmental conditions for *H. capsulatum* chitinase expression or a yeast-phase-specific chitinase.

#### Enzymatic Activities of H. capsulatum Chitinases

**Fig. 6.**
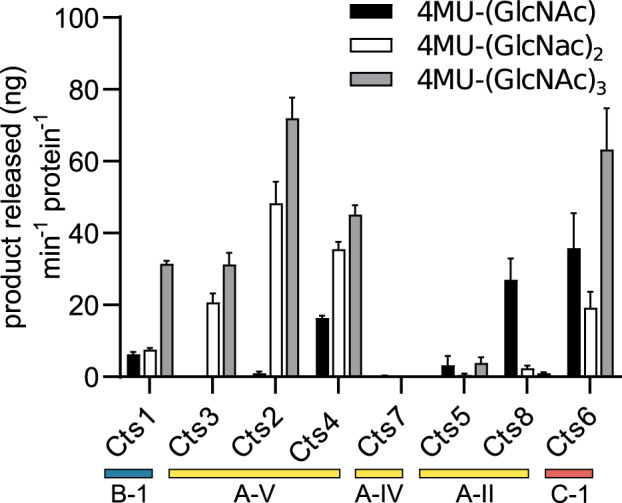
Enzymatic activity of *Histoplasma capsulatum* chitinases. The enzymatic activities of purified *H. capsulatum* chitinases were tested using fluorigenic GlcNAc oligomers linked to 4-methylumbelliferone (4MU) by a glycosidic bond. Chitinase activity that hydrolyzes this linkage liberates 4-MU which was detected by fluorescence. Substrates tested were 4MU-(GlcNAc) (black bars), 4MU-(GlcNAc)_2_ (white bars), 4MU-(GlcNAc)_3_ (gray bars). The exochitinase activity, N-acetyl-glucosaminidase, processively removes GlcNAc saccharides from the nonreducing end resulting in hydrolysis of all substrates. Chitobiosidase activity (release of chitobiose from the nonreducing end) is detected as hydrolysis of 4MU-(GlcNAc)_2_. Endochitinase activity generates fluorescence from 4MU-(GlcNAc)_2_ and 4MU-(GlcNAc)_3_. Chitobiase activity (hydrolysis of the disaccharide chitobiose) generates fluorescence only from the chitobiose mimic 4MU-(GlcNAc). Activity rates were calculated as nanograms of hydrolysis product (4MU) released per minute per nanogram of *H. capsulatum* protein. Data represent the average activity ± SD of replicates (*n* = 3). GH18 subclades to which each Cts protein belongs are indicated (A, yellow; B, blue; and C, red).

To determine if the clades represented by the *H. capsulatum* chitinases (Cts proteins) correspond to different enzyme activities as suggested by previous phylogenetic studies, all eight *H. capsulatum* chitinases were purified and tested for chitin degradation profiles. Three artificial substrate mimics were used to determine the specificity of each purified chitinase enzyme (fig. 6): *N*-acetyl-glucosaminidase exochitinase activity (liberation of 4-methylumbelliferone [4-MU] from all substrates including sequential hydrolysis of GlcNAc units from the reducing end of oligosaccharides), endochitinase activity (liberation of 4-MU from oligosaccharides with at least two GlcNAc units), chitobiosidase activity (hydrolysis of the disaccharide chitobiose from the reducing end thus liberating 4-MU from 4MU-(GlcNAc)_2_), and chitobiase, which hydrolyzes only the disaccharide chitobiose (e.g., hydrolysis only of the glucosidic bond in 4-MU-GlcNAc). The B-clade chitinase Cts1 exhibited endochitinase activity (fig. 6) consistent with the proposed activity suggested by the more open-channel B-clade enzyme structure (Hartl et al. 2012). The A-V chitinases (Cts2, Cts3, and Cts4) also showed endochitinase activity and chitobiosidase function consistent with hydrolysis of internal glucosidic linkages. Cts4 also had a low level of exochitinase activity similar to *N*-acetyl-glucosaminidases suggesting neofunctionalization after duplication from Cts2. Of the other A-clade proteins, only Cts8 (an A-II subclade protein) exhibited significant chitinase activity, which was consistent with chitobiase activity (fig. 6). The other A-II subclade protein, Cts5 had detectable but extremely poor activity on any substrate. Cts7 (subclade A-IV), lacked any detectable activity (fig. 6), consistent with the lack of active site residues in the Cts7 protein (fig. 4). Cts6, *H. capsulatum*’s only C-clade protein (C-1 subclade) hydrolyzed all substrates consistent with *N*-acetyl-glucosaminidase exochitinase activity (fig. 6).

## Discussion

### Previous Chitinase Ontologies Are Largely Robust to Increased Sampling, but the A Clade Is Polyphyletic

Our analyses support the existence of two major classes of chitinases defined by an ancient divergence between B and AC seen in previous phylogenetic analyses. We also recapitulate the previous subclades with varying degrees of support (fig. 1 and table 1). However, we find that there is not a single A clade, because C clade chitinases are more closely related to A-II chitinases. The divergence of the A-IIIs and the close clustering of the A-II subclade with the C clade suggest the definition of A and C clades could use revision. Since A clade chitinases are not monophyletic, either C clade chitinases should be subsumed into a larger A clade, or alternately, the A clade chitinases could be split into multiple new clades informed by additional gene architecture and enzymatic activity investigations. Previous work has supported both an independent C clade (Seidl et al. 2005; Karlsson and Stenlid 2008; Alcazar-Fuoli et al. 2011) and a C + A-II clade (Karlsson and Stenlid 2009) that is consistent with our analyses. However, only the present analysis strongly supports the divergence of the A-III clade from the rest of the A subclades and the C clade, perhaps because the limited sample of A-III chitinases in previous analyses (<10 members) did not reflect overall A-III diversity. Our analysis suggests additional fungal chitinases need to be characterized in order to accurately describe functional diversity among chitinases and thereby more accurately inform the designation of clades and subclades. Additionally, the location of the LysM domain has been used to distinguish the C clade from the A clade (Gruber, Vaaje-Kolstad, et al. 2011; Gruber and Seidl-Seiboth 2012); however, in our analyses, LysM domains are found in the C-II subclade as well as some A-V chitinases, including the *H. capsulatum* Cts3 (fig. 4). Furthermore, C-I subclade chitinases often lack a detectable LysM domain (e.g., H*. capsulatum*’s Cts6). Thus, the presence of LysM is not a C-clade-defining feature as originally proposed. Additionally, the A clade was generally thought to be lacking in CBDs (Hartl et al. 2012); however, CBDs are widespread in the A-II subclade (fig. 3), further supporting the need to redefine the A and C clades.

### Species Representation within Chitinase Subclades Are Explained by Phylogeny and Ecology

The B versus AC split appears to be an early divergence in chitinases, preceding the origin of fungi. For the B clade (GH18 class III) chitinases, previous analyses suggest that B-Vs were the first to diverge after the fungal B chitinases split from bacterial chitinase (Karlsson and Stenlid 2009). Of the AC (Class V) chitinases, subclade A-V is the most widespread throughout the taxa, with members found in early-diverging fungi, Basidiomycota, and Ascomycota, suggesting it maintains a core function in fungal physiology. In contrast, the A-III subclade is greatly reduced in Ascomycota, as compared with early-diverging fungi and Basidiomycota, suggesting A-III functions may be conditionally dispensable in Ascomycota. In general, the Pezizomycotina (Ascomycota), particularly Leotiomycetes, Sordariomycetes, Eurotiomycetes, and Dothideomycetes, have chitinases from the greatest diversity of subclades. Genomes of early-diverging fungi generally contain few chitinases, and these are mostly from the C-II, A-V, and particularly the A-III subclades. *Rozella allomycis* is an early-diverging species that lacks chitin, but maintains a single A-V chitinase that may play a role in its obligate endoparasitism of chytrid fungi (Jones et al. 2011).

Clearly restricted taxonomic distributions of certain chitinase subclades suggest they may be ancestrally orthologous. For example, the B-IV subclade is strictly limited to the Saccharomycetales. However, most B-IV characterized functions (table 2) relate to fungal cell division and morphology (Kuranda and Robbins 1991; Selvaggini et al. 2004; Colussi, Specht, and Taron 2005; Hurtado-Guerrero and van Aalten 2007). As cell division is fundamental, it would be unusual that this function itself is performed only by chitinases in Saccharomycetales. The constitutive expression of *H. capsulatum*’*s* B-clade chitinase, and the GPI-attachment motif suggestive of anchoring at the cell surface, supports a generalized function for B clade chitinases in fungal cell division and growth. The B-III subclade may represent the Basidiomycota-specific B chitinases, but as delimited it also includes Mucorales chitinases, which may reflect uncertainty in deep branching order, or loss of an Ascomycota paralog. B-I and B-II chitinases are limited to the Pezizomycotina and may represent a taxon-specific radiation of B chitinases.

**Table 2.** Enzymatically Characterized Proteins.

Subclade	Taxon	Protein	Organism	Activity^a^	Reference
A-II	Eurotiales	Cfu1	*Aspergillus niger*	Exochitinase (NAG)	van Munster et al. (2012)
	Onygenales	Cts5	*Histoplasma capsulatum*	Exochitinase (NAG)	This study
	Onygenales	Cts8	*Histoplasma capsulatum*	Chitobiase	This study
A-III					
A-IV	Onygenales	Cts7	*Histoplasma capsulatum*	(No activity)	This study
A-V	Eurotiales	ChiB1	*Aspergillus fumigatus*	Endochitinase	Jaques et al. (2003)
	Hypocreales	Ech42/Chit42	*Trichoderma harzianum*	Endochitinase/chitobiosidase	Haran et al. (1995)
	Onygenales	CiX1/Cts1	*Coccidioides immitis*	Endochitinase/chitobiosidase	Fukamizo et al. (2001)
	Onygenales	Cts3	*Histoplasma capsulatum*	Endochitinase/chitobiosidase	This study
	Onygenales	Cts2	*Histoplasma capsulatum*	Endochitinase/chitobiosidase	This study
	Onygenales	Cts4	*Histoplasma capsulatum*	Endochitinase/chitobiosidase/(weak exochitinase)	This study
	Hypocreales	CHIT42	*Metarhizium anisopliae*	Endochitinase/exochitinase (NAG)	Baratto et al. (2003)
C-I	Onygenales	Cts6	*Histoplasma capsulatum*	Exochitinase (NAG)	This study
C-II					
B-I	Eurotiales	ChiA1	*Aspergillus fumigatus*	Not well defined	Rush et al. (2010)
	Hypocreales	Ech30	*Trichoderma atroviride*	Endochitinase	Hoell et al. (2010)
	Onygenales	Cts1	*Histoplasma capsulatum*	endochitinase	This study
B-II	Hypocreales	Chit33	*Trichoderma harzianum*	Endochitinase/chitobiosidase	Haran et al. (1995); Boer et al. (2007)
B-III					
B-IV	Saccharomycetales	Cts1	*Sacharomyces cerevisiae*	Not well defined	Kuranda and Robbins (1991); Hurtado-Guerrero and van Aalten (2007)
	Saccharomycetales	Chit2	*Candida albicans*	Not well defined	Selvaggini et al. (2004)
	Saccharomycetales	Chit3	*Candida albicans*	Not well defined	Selvaggini et al. (2004)
	Saccharomycetales	Cts1	*Kluyveromyces lactis*	Endochitinase	Colussi et al. (2005)
B-V					
“B”	Hypocreales	Chit1	*Beauveria bassiana*	Endochitinase	Fang et al. (2005); Fan et al. (2007)
D-like	Hypocreales	CHIT30	*Metarhizium anisopliae*	Endochitinase/chitobiosidase/exochitinase (NAG)	Pinto et al. (1997); da Silva et al. (2005)

a Enzymatic activity: hydrolysis of (GlcNAc)_n_. Exochitinase (NAG): N-acetyl-glucosaminidase activity where *n* ≥ 2 or greater. Endochitinase: *n* > 2. Chitobiosidase: *n* > 2 with liberation of the disaccharide chitobiose (GlcNAc)_2_. Chitobiase: *n* = 2. Not well defined: limited substrates tested (e.g., only [GlcNAc]_n_ with *n* > 2) which does not exclude N-acetyl-glucosaminidase (exochitinase) from endochitinase activity.

The fungal ecological diversity represented among chitinase clades is complex, precluding simple ecological explanations for differences among chitinase repertoires; however, the variability in the number of chitinases in species is consistent with some expectations. For example, the genomes with the highest number of chitinases include fungi with predominantly insect and fungus-derived nutrition like Basidiobolus (Tabima et al. 2020) Cordyceps, and Trichoderma (supplementary data 7, Supplementary Material online), but also wood-decay fungi like Ganoderma and Auricularia, which may use additional chitinases in competition or secondary decomposition. In contrast, we see the fewest chitinases (≤1 per sequenced genome) in fungi that are sequestered from the open environment or obligately associated with plants or animals. For example, few chitinases are produced by Rozella (endoparasites), Neocallimastigomycota (obligate gut rumen symbionts), Glomerales (arbuscular mycorrhizae), and Malassezia (skin commensals). We identified no GH18 chitinases in the obligate intracellular animal parasites Microsporidia, and the GH18 function may have been subsumed by their recently described GH19 chitinases (Han et al. 2016).

Although it was not feasible to perform a comprehensive analysis of individual molecular evolution events among fungal chitinases in this data set, there is evidence for a significant role of HGT in their diversification, and these events suggest ecological roles for the transferred genes (supplementary fig. S2 and supplementary data 5, Supplementary Material online). For example, we observed multiple HGT events and gene family expansions among insect-associated fungi. As insect exoskeletons are a major source of chitin (Tharanathan and Kittur 2003), the acquisition and expansion of chitinases might facilitate greater chitinase production or enable degradation of diverse forms of arthropod chitin under diverse environmental conditions or pathogen relationships (Karlsson and Stenlid 2009). LysM domain-containing chitinases are distributed among plant pathogenic, insect parasitic, and saprotrophic fungi.

LsyM domains have been shown to bind chitin although carbohydrate-binding specificities are not well-described and their role in most fungi is not well understood (Gruber, Vaaje-Kolstad, et al. 2011). In our analysis, the LysM domain, although largely corresponding to the C-II clade, is not a clade-defining characteristic, making prediction of the functional role of such chitinases difficult (fig. 3). Some LysM-containing proteins have also been shown to bind fungal chitin, contributing to fungal evasion of host plant immunity (Bolton et al. 2008; de Jonge and Thomma 2009). One chitinase in the single Chytrid genome analyzed, *Spizellomyces punctatus*, which may be a decomposer of arbuscular mycorrhizal fungi (Paulitz and Menge 1984) contains a large number of LysM domains. However, although there are many plant and animal pathogens with LysM-containing chitinases, there does not appear to be a strong ecological association (Stergiopoulos et al. 2012).

### Evolution of the *H. capsulatum* Chitinases Indicates a Degree of Differentiation and Expansion That Is Reflected in Fungal Chitinases in General

Placing *H. capsulatum* in context of the other Onygenales analyzed, *H. capsulatum* was the only genus out of the sequence Onygenales that contained A-II subclade members. One of the most interesting features of *H. capsulatum* chitinase evolution is the presence of two chitinases in this A-II subclade. A-II subclade members are not widespread in fungal taxonomy; they are almost exclusively found in the Leotiomyceta (subdivision of the filamentous Pezizomycotina). A-II chitinases are found in Eurotiomycetes, Sordariomycetes, Dothideomycetes, and Leotiomycetes. In the Eurotiomycetes, Eurotiales, Onygenales, and Chaetothyriales contain A-II subclade members. However, *H. capsulatum* is the only species in Onygenales currently known to contain this subclade, although we cannot rule out the possibility that other A-II subclade proteins might be found in Onygenales species yet to be sequenced. This suggests these chitinases are ancestral to the Leotiomyceta, but they have been largely lost in the Onygenales. Therefore, these may have a specific function that is necessary for *H. capsulatum* biology that is lacking in many other Onygenales.

The B-clade chitinase in *H. capsulatum* (Cts1) belongs to the B-I clade. The other Onygenales tend to contain B-II subclade members that *H. capsulatum* lacks. In addition, the C clade chitinases are expanded in multiple other Onygenales members but are restricted to one chitinase in *H. capsulatum* (Cts6). This is most obvious in *Microsporum canis* with nine C-I and four C-II members. However, upon closer examination, only three of each subclade are predicted to be active (contain the active site residues) indicating that this larger clade representation may not be as functionally extreme as otherwise indicated.

In terms of other members of Eurotiomycetes, the Onygenales and Eurotiales show matching patterns of chitinases distribution in the subclades. Chaetothyriales differ in that they contain A-III subclade chitinases members but are lacking B-II or C clade members seen in the other Eurotiomycetes. When comparing the Eurotiomycetes to other Pezizomycotina, Saccharomycetes is rather divergent. It is the only one to contain a B-IV domain, and is the only order from the Pezizomycotina to be missing a B-V or C-I subclade chitinase from the Pezizomycotina. Saccharomycetes are limited to the B-IV, C-II, A-IV, and A-V subclades, which is less diversity seen in other Pezizomycotina. Sordariomycetes and Eurotiomycetes had identical patterns of subclade distribution, missing the A-III proteins. Leotiomyceta members (excluding Xylonomycetes) have A-II, B-I, and C-II subclade proteins. The A-IV clade is missing in Orbiliomycetes, whereas C-I is missing in Xylonomycetes and Pezizomycetes. The A-V subclade is conserved in all Pezizomycotina.

### *Histoplasma capsulatum* Chitinase Expression and Functional Differences Suggest Complementary Functional Roles

*Histoplasma capsulatum* expression data allowed for an initial look at how multiple chitinases could potentially play different roles. Most *CTS* genes did not exhibit significant transcriptional regulation by morphological state or nutritional conditions including the presence of chitin. For such seemingly constitutively expressed *CTS* genes, they may be regulated by other unknown environmental conditions or the Cts functions may be regulated posttranscriptionally.

Cts1 is secreted and contains a GPI-attachment motif indicating that it is likely anchored at the fungal cell wall for cell wall remodeling or modification during cell division and growth. Cts1 had a low, but generally constant level of expression in all growth conditions consistent with an essential function in fungal growth. As transcriptional data were not collected on synchronized stages of division or morphology, it is possible the low expression is a result of a mix of cells at different stages of replication with high and low expression. The finding that Cts1 is an endochitinase active only on larger oligosaccharides (fig. 6) rather than a processive exochitinase fits with a putative role in remodeling, but not complete dismantling, of chitin in the fungal cell wall.

Cts3 was the only chitinase that was induced in the mycelial phase of *Histoplasma*, supporting a functional role in the mycelial state of this organism, which is an environmental saprotroph in contrast to the yeast phase that is parasitic for animals. As Cts3 lacks a secretion signal, this may suggest that Cts3 plays an intracellular role in cell division, septa formation, or in the preparation of the cell wall for formation of mycelial-specific structures (e.g., conidiophores). The chitobiosidase activity of Cts3 suggests the existing cell wall must be extensively altered for such structures. Chitinases are generally expanded in fungi characterized by filamentous growth and are few in yeast-form fungi like *S. cerevisiae*, supporting the hypothesis that hyphal growth or reproductive morphologies requires multiple different chitinases (Karlsson and Stenlid 2008). As Cts3 is the only *H. capsulatum* chitinase with a LysM domain, this may further indicate that Cts3 activity is directed at “self” chitin (e.g., during formation of hyphal wall structures) similar to the role of LysM-proteins in binding fungal pathogen chitin to prevent plant immune responses. *Histoplasma capsulatum* dimorphism is strictly regulated (Edwards et al. 2013), primarily controlled by the Ryp transcription factors (Shen and Rappleye 2017; Sil 2019), whereas other common fungal transcription factors, such as the APSES family, appear to have very limited function in dimorphism (Longo et al. 2018). Therefore, any chitinases necessary for hyphal growth or mycelial structures should be closely linked to transcriptional regulation of the mycelial state.

*CTS2*, which encodes another A-V subclade chitinase, has consistently higher expression among all *H. capsulatum* chitinase genes. This suggests that under the conditions tested Cts2 may be the general functional chitinase, whereas the other related chitinase, Cts4, is either simply less used or used under specific, unknown conditions. Other *H. capsulatum* chitinases show low or highly variable expression among diverse conditions precluding specific hypotheses as to their biological roles based on gene expression.

As *H. capsulatum* is the only detectable Onygenales species with A-II chitinases, the secreted Cts5 and Cts8 chitinases are of particular interest. Cts8 hydrolysis activity is consistent with that of a chitobiase, an enzyme that specifically hydrolyzes the disaccharide chitobiose which is produced by the activity of chitobiosidases. This may indicate that the primary role of Cts8 is to further hydrolyze disaccharides in the extracellular environment (such as those produced by other chitinases) into GlcNAc monomers. We hypothesize that Cts8 thus plays a nutritional role by liberating hexosamines from other chitin hydrolysis products. Alternatively, generation of GlcNAc monomers from environmental chitin degradation products (i.e., chitobiose) may serve a signaling function since adoption of the mycelial state is potentiated by free GlcNAc but not glucosamine (Gilmore et al. 2013). The other A-II *Histoplasma* chitinase, Cts5, has very minimal activity, which along with the lack of any detectable expression suggests that this chitinase may no longer have a functional role.

*CTS7* expression is very low to undetectable, and Cts7 lacks any chitinase activity suggesting that it also may no longer serve a functional role in *H. capsulatum* biology. Consistent with this, Cts7 also lacks a signal sequence for secretion unlike the majority of the other chitinases. However, at this time a nonchitin-hydrolysis role for Cts7 cannot be ruled out.

Cts6 is the most unique of *H. capsulatum*’s chitinases having multiple domains and an increased size. Cts6 is the only C-clade chitinase produced by *H. capsulatum*, and the expression pattern of *CTS6* suggests that its transcription increases in the presence of exogenous chitin. Together these characteristics suggest that Cts6 may be suited for hydrolysis of diverse chitin substrates in the environment. Consistent with a role in the degradation of environmental chitin, Cts6 is secreted and has *N*-acetyl-glucosaminidase activity (fig. 6), an exochitinase function which would enable it to processively hydrolyze chitin polysaccharides.

### *Histoplasma capsulatum* Chitinase Specificity Demonstrates the Difficulty in Using Phylogeny for Activity Prediction

Determination of the chitinase activities of the eight *H. capsulatum* chitinases highlights the limitations of previous phylogenetic analyses to predict enzymatic activities. Few fungal chitinases have been enzymatically characterized (table 2) and the characterization is sometimes not complete. The multiple A-V *H. capsulatum* chitinases illustrate how functional variation can be present even within subclades, such as the exochitinase activity of Cts4 in addition to the endochitinase and chitobiosidase activity of its closest paralog Cts2. When compared with some other characterized A-V clade members, we see a similar pattern of multiple and complex activities (table 2). The demonstrated endochitinase activity with Cts2 and Cts3 is inconsistent with the previous prediction that A clade members are characterized by exochitinase activity (Duo-Chuan 2006; Seidl 2008; Hartl et al. 2012). However, this subclade also shows a strong presence of chitobiosidase activity (table 2). The A subclades might have functionally diverged, which may be reflected in the potential polyphyletic phylogeny of the A clade chitinases. For example, the characterized members of the A-II subclade have varied activities, not the previously suggested restriction to exochitinase activity (table 2). In addition, the A-III subclade is lacking in enzymatically characterized members, and the A-IV only has one characterized member (Cts7 from this study), which does not have any enzymatic activity at all. As the A subclades are more variable in enzymatic activity than expected and the A-III subclade is so divergent from the other AC subclades, these chitinases need further study to uncover how the A subclades have diverged and how that might influence their activities.

This study is the first report of the enzymatic activity of a C clade member (table 2). The hydrolysis profile of *H. capsulatum* Cts6 is consistent with an *N*-acetyl-glucosaminidase as the successive hydrolysis of GlcNAc from oligosaccharides enables this enzyme to hydrolyze all substrates tested (fig. 6).

In contrast to the AC clade, the B clade functional data are much more consistent with predictions, with characterized members having endochitinase or chitobiosidase activity (Duo-Chuan 2006; Seidl 2008; Hartl et al. 2012). However, subclades B-III and B-V have not been extensively investigated to determine the universality of the prediction for B clade enzymes (table 2). In addition, some of these functionally characterized enzymes have not been extensively studied for substrate preference and thus a strict categorization is premature.

The large increase in available fungal genomes is improving our understanding of the evolutionary relationships among fungal chitinases. As demonstrated in this study, combination of phylogenetics with empirical characterizations, including determination of enzymatic activities, further refines the predictive power of fungal chitinase phylogenetic classifications. Using this fungal chitinase phylogenetic framework, future studies to define the functional roles of enzymes using the increased feasibility of molecular genetic tools for fungi (Wang et al. 2017; Raschmanová et al. 2018; Song et al. 2019) will connect evolution of chitinases to their individual and specific roles in fungal biology.

## Materials and Methods

### Phylogenetic Analyses

Putative chitinases were retrieved from 373 published fungal genomes, including six *Histoplasma* genomes (supplementary data 1, Supplementary Material online) obtained from the Joint Genome Institute (JGI) website and NCBI. Proteomes were independently mined for glycosyl hydrolase 18 (GH18)-containing proteins (El-Gebali et al. 2019) using HMM search (Eddy 2009). A CDD search was then performed on proteins containing GH18 domains to identify additional carbohydrate-binding domains (Marchler-Bauer et al. 2017) and LysM domains. Alignments of the GH18 domains and LysM domains were performed by HMMalign which uses a Viterbi algorithm to align each sequence to the given HMM (Eddy 2009). Sequences were removed if they were missing alignment between positions 87–238. Poorly aligned characters were removed using trimAl (v. 1.4) (Capella-Gutiérrez et al. 2009) with a gap threshold of 0.01.

Phylogenetic analysis of the GH18 domains was performed using maximum-likelihood methods using VT+F+G4 (GH18) or WAG+R5 (LysM) model (automatically determined) in IQ-TREE (Nguyen et al. 2015). This tree was used to determine the sequences in the AC clades and the B clade. These sequences were then split into separate phylogenetic analyses again using maximum-likelihood methods (model WAG+G4 for AC tree and PMB+F + I+G4 for 4B tree as automatically determined) implemented in IQ-TREE (Nguyen et al. 2015). Statistical support for all IQ-TREEs was assessed by Ultrafast bootstrap analysis using 1,000 replicates (Minh et al. 2013). An ultrafast bootstrap value of ≥95 was considered a strongly supported branch. Parameters of additional phylogenetic analyses in IQ-TREE to assess support for HGT events, including topology tests using the approximately unbiased test (Shimodaira 2002) implemented in IQ-TREE are in supplementary data 5, Supplementary Material online. In addition, maximum-likelihood methods were implemented in RAxML v.(SSE3) (Stamatakis 2014), 100 alternative runs on 100 distinct trees, bootstrapping (100) and best-scoring ML in 1 run and GAMMA models autodetermined (WAG for AC and PMB for B). Statistical support for RAxML trees was assessed by rapid bootstrapping, where nodes receiving ≥60% of bootstraps were considered supported.

### Chitinase Gene Expression

*Histoplasma capsulatum CTS* gene transcription was determined by quantitative RT-PCR (qRT-PCR). The WU15 strain of *H. capsulatum*, a uracil auxotroph of the North America 2 clade was grown on HMM (Worsham and Goldman 1988) or Sabouraud’s Dextrose Agar (SDA) media supplemented with 100 µg/ml uracil and solidified with 0.6% agarose. For tests of chitin induction of *CTS* gene transcription, colloidal chitin (prepared from shrimp chitin; Rodriguez-Kabana et al. 1983) was added to media (1.2% final concentration). Media were inoculated with *H. capsulatum* cells and incubated at 25° (mycelial culture) for 4 weeks or 37° (yeasts culture) for 5–7 days. *Histoplasma capsulatum* cells were scraped from the solid media and collected by centrifugation (5 min at 2,000 × g). RNA was isolated from fungal cells by mechanical disruption with 0.5 mm glass beads, extraction with RiboZol (Amresco), and purification using an affinity column (Direct-zol RNA MiniPrep Plus; Research Products International). Following DNA removal with DNase Invitrogen, RNA was reversed transcribed (Maxima reverse transcriptase; Thermo Scientific) primed with random pentadecamers. Quantitative PCR was carried out using *CTS* gene-specific primer pairs (supplementary data 8, Supplementary Material online) with SYBR green-based visualization of product amplification (Bioline). Changes in *CTS* gene transcript levels relative to Actin (*ACT1*) and Ribosomal Protein S15 (*RPS15*) were determined using the ΔΔCt method (Schmittgen and Livak 2008) after normalization of cycle thresholds to *ACT1* and *RPS15* mRNA levels. RNAs were prepared from three biological replicates for each media/condition. Significant differences in expression (*P* < 0.05) were determined by Student’s *t*-test between paired samples differing by a single parameter. Samples with transcripts below the detectable limit were set to −12.00 ΔΔCt for analysis purposes.

### Purification of *H. capsulatum* Chitinases

Chitinases were purified from transgenic *H. capsulatum* yeasts overexpressing each protein. Chitinase-encoding genes were amplified by PCR from wild-type *H. capsulatum* G217B genomic DNA and cloned into the *H. capsulatum* expression vector pAG38 placing the gene under transcriptional control of the *H2B* constitutive promoter and fusing the chitinase to a C-terminal hexahistidine tag. For Cts1, sequences encoding the putative GPI-attachment site (nucleotides 2,141–2,215) were removed to permit recovery of soluble protein. Overexpression plasmids were transformed into *H. capsulatum* WU15 yeasts by *Agrobacterium*-mediated transformation (Zemska and Rappleye 2012) and transformants selected by uracil prototrophy. Transformants were screened by immunoblotting of culture filtrates and cellular lysates for the hexahistidine tag (GnScrpt antibody A00186 to 6× histidine). Chitinase-expressing transformants were grown in liquid HMM until stationary phase and the yeasts separated from the culture supernatant by centrifugation (5 min at 2,000 × g). For Cts1, Cts2, Cts4, Cts5, Cts6, and Cts8, culture filtrates were prepared by filtration of the supernatant (0.45 μm pore; Millipore), concentrated 100-fold by ultrafiltration (10 kDa MWCO; Whatman), and the proteins exchanged into phosphate-buffered saline (PBS). For Cts3 and Cts7, which lack secretion signals, lysates were prepared from the yeast cells by suspension of yeasts in PBS and mechanical breakage with 0.5 μm diameter glass beads. Debris was removed from the cellular lysate by centrifugation (10 min at 12,000 × g). Hexahistidine-tagged chitinase proteins were purified from the concentrated culture filtrates or cellular lysates by metal affinity chromatography (HisPur Co^2+^ Resin, Thermo Fisher Scientific) and the chitinase-containing elution fractions exchanged into PBS by ultrafiltration. Resultant protein concentrations were determined using a Bradford assay (Sigma-Aldrich) and purity of the protein preparation determined by SDS–PAGE followed by silver staining. Purified proteins were stored at −20 °C in 50% glycerol.

### Chitinase Activity and Specificity Determination

Chitinase enzymatic activities were determined via a fluorimetric chitinase assay kit (Sigma-Aldrich-CS1030). Three artificial substrate mimics, 4-methylumbelliferyl-*N*-acetyl-β-d-glucosaminide (4MU-(GlcNAc)), 4-methylumbelliferyl *N*,*N*′-diacetyl-β-d-chitobioside (4MU-(GlcNAc)_2_), and 4-Methylumbelliferyl β-d-*N*,*N*′,*N*″-triacetylchitotriose (4MU-(GlcNAc)_3_) were used to determine the activity of each chitinase. Hydrolysis of each substrate releases 4-methylbelliferone, the fluorescence of which was measured using a plate reader (360-nm excitation and 450-nm emission; BioTek Synergy 2). For biochemical reactions, varying amounts of purified chitinases were added to 0.5 mg/ml substrate in reaction buffer and incubated at 37 °C. Reactions were monitored by endpoint fluorescence at 60 min. Data are reported as nanograms of 4-methylumbelliferone released per minute per nanogram of purified chitinase. The equation of the best-fit line to the data was computed and compared with a standard curve of 4-methylumbelliferone. Activity assays were performed in triplicate.

## Supplementary Material

Supplementary data are available at *Molecular Biology and Evolution* online. Other data are available by request of the authors.

## Supplementary Material

msaa293_Supplementary_Data
