# Supplementary material for: Diversification of Fungal Chitinases and Their Functional Differentiation in Histoplasma capsulatum
Source: Mol Biol Evol. 2020 Nov 13;38(4):1339–55. doi: 10.1093/molbev/msaa293 (PMC8042737; doi:10.1093/molbev/msaa293)

Supplemental Figure S1. Phylogenetic analysis of fungal GH18 domains

An unrooted phylogenetic tree depicts relationships among 3,888 fungal chitinase proteins based on alignment of their glycosyl hydrolase (GH18) domains. Trees were built using maximum-likelihood methods (IQ-Tree). Major groups corresponding to the previously named A, B, and C clades are indicated by color (yellow, blue, red, respectively). Clades comprised of bacterial-like GH18 proteins are indicated (orange).

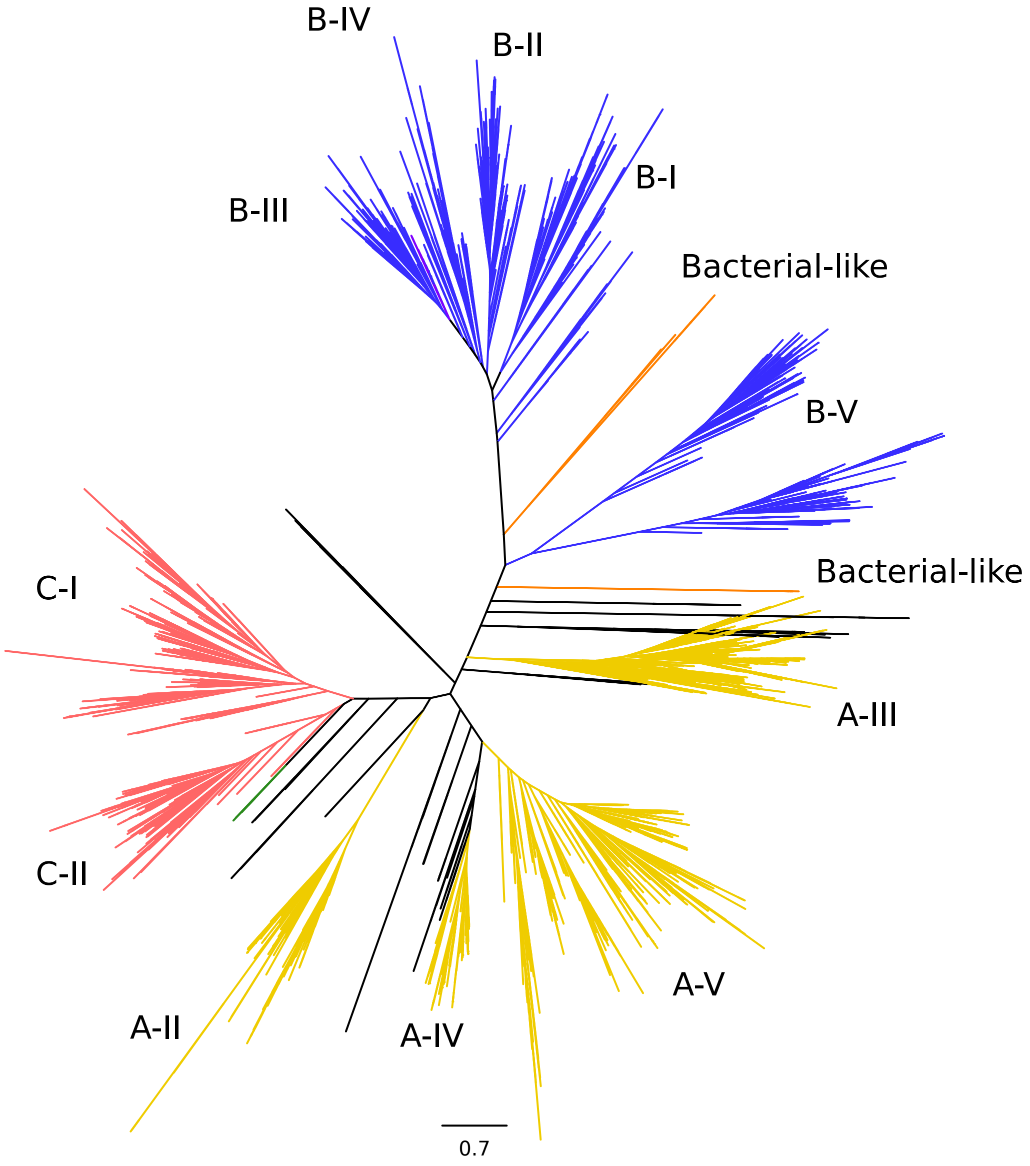

Supplemental Figure 2. Detection of potential horizontal gene transfer occurrances
Maximum Likelihood phylogenies (IQ-tree) to infer origins of potential HGT chitinases (red text). (**A**) The A clade of chitinases from arthropod pathogens in Hypocreales occupy an uncertain position among diverse bacteria. (**B**) The A clade of mostly Hypocreales chitinases are placed within Streptomyces (Actinobacteria). (**C**) Two clades of fungal chitinases occupy separate parts of the phylogeny of D-like chitinases from Actinobacteria. (**D**) The A clade of Kickxellales chitinases also contains sequences from multiple hypocrealean insect pathogens. (**E**) The B-V chitinase from Basidiobolus meristosporus (Zoopagomycota) is placed in Agaricomycetes (Basidiomycota). (**F**) The C-II chitinase in Panaeolus cyanescens (Agaricales) is sister to a chitinase in Uncinocarpus reesei (Onygenales). Support values indicate percentage of rapid bootstraps.

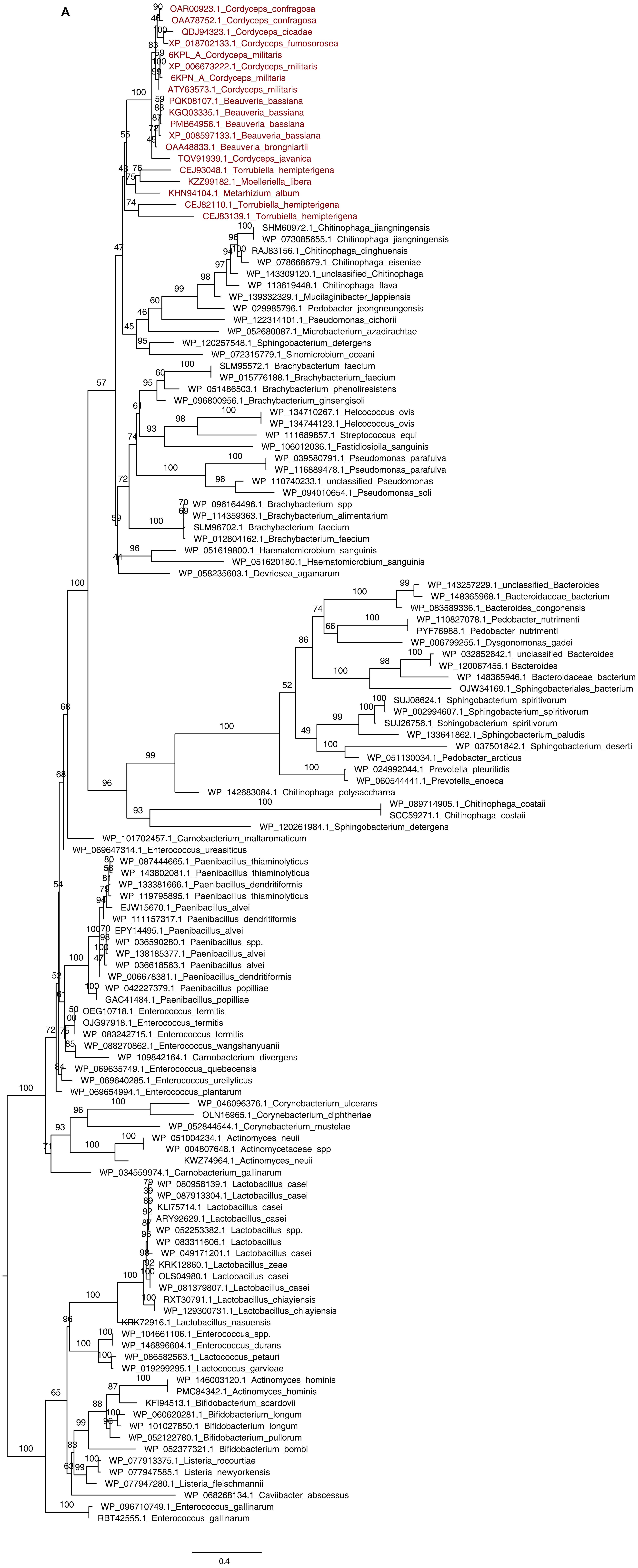

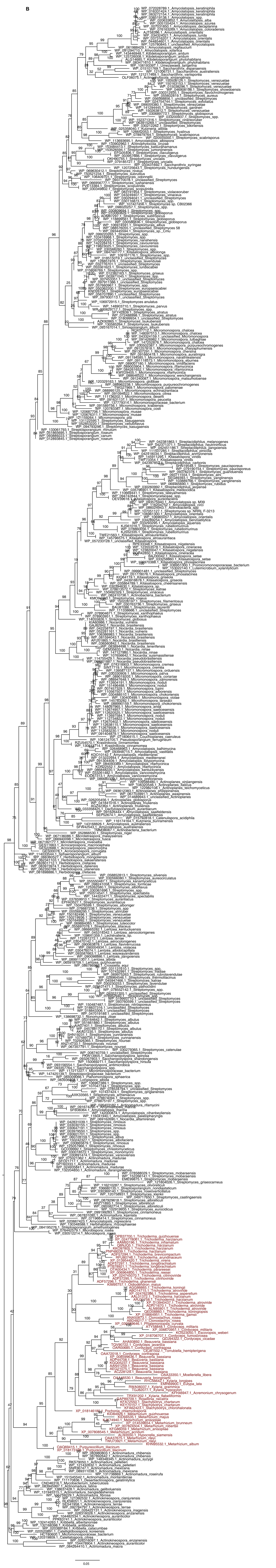
B
0.05

c

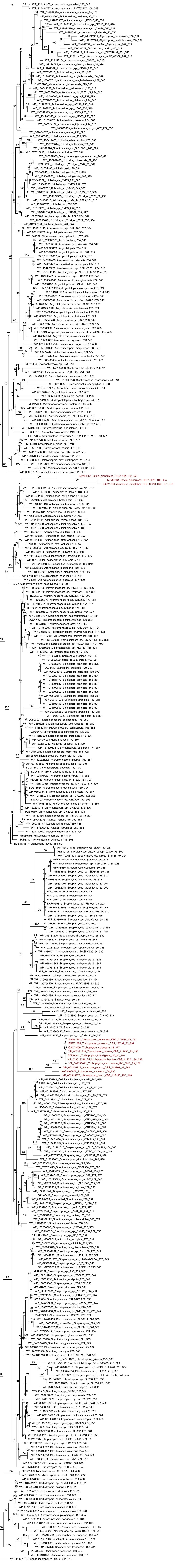

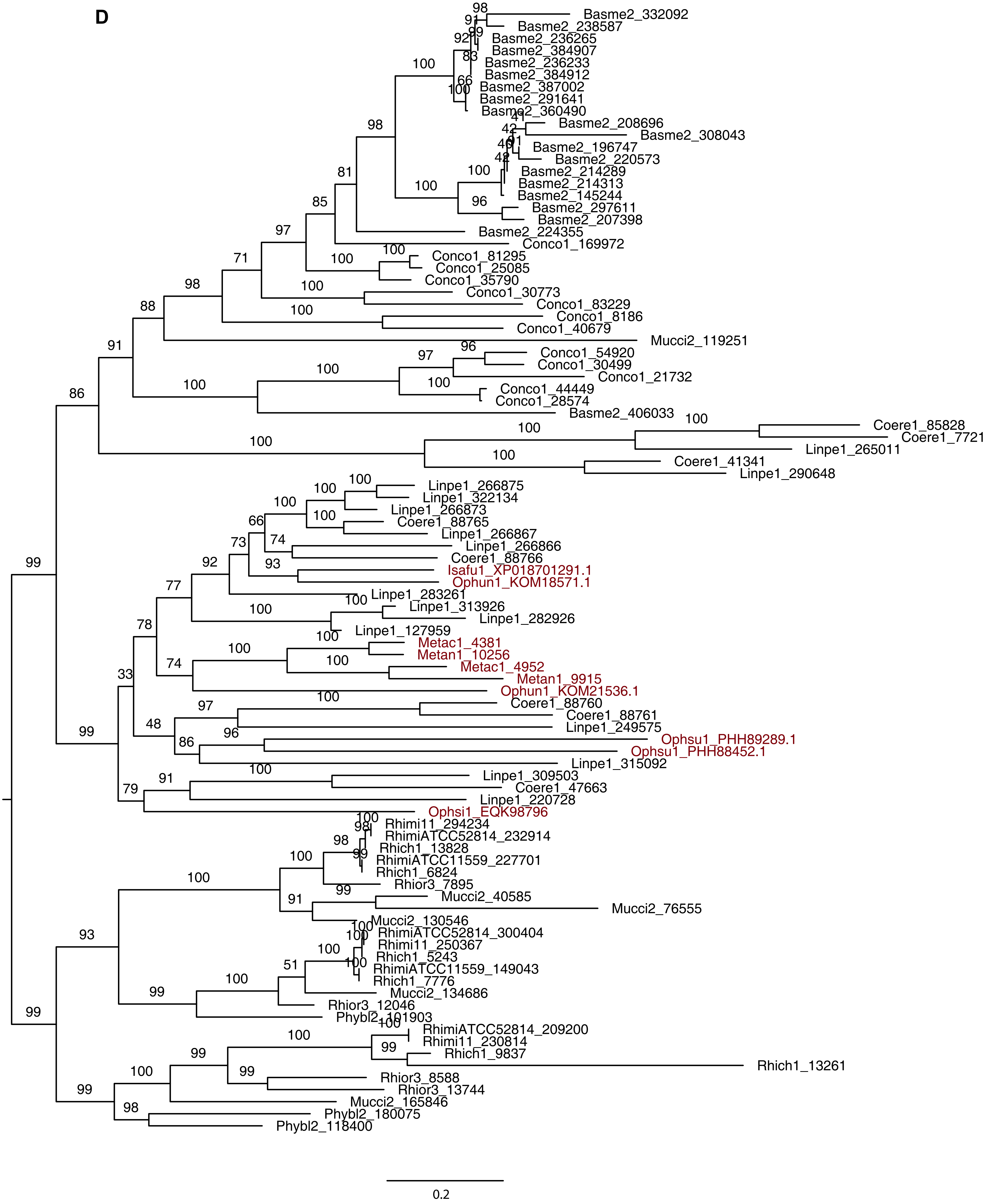
D
Basme2_332092
Basme2_238587
Basme2_236265
Basme2_384907
Basme2_236233
Basme2_384912
Basme2_387002
Basme2_291641
Basme2_360490
Basme2_208696
Basme2_308043
Basme2_196747
Basme2_220573
Basme2_214289
Basme2_214313
Basme2_145244
Basme2_297611
Basme2_207398
Basme2_224355
Conco1_169972
Conco1_81295
Conco1_25085
Conco1_35790
Conco1_30773
Conco1_83229
Conco1_8186
Conco1_40679
Mucci2_119251
Conco1_54920
Conco1_30499
Conco1_21732
Conco1_44449
Conco1_28574
Basme2_406033
Coere1_85828
Coere1_7721
Linpe1_265011
Coere1_41341
Linpe1_290648
Linpe1_266875
Linpe1_322134
Linpe1_266873
Coere1_88765
Linpe1_266867
Linpe1_266866
Coere1_88766
Isafu1_XP018701291.1
Ophun1_KOM18571.1
Linpe1_283261
Linpe1_313926
Linpe1_282926
Linpe1_127959
Metac1_4381
Metan1_10256
Metac1_4952
Metan1_9915
Ophun1_KOM21536.1
Coere1_88760
Coere1_88761
Linpe1_249575
Ophsu1_PHH89289.1
Ophsu1_PHH88452.1
Linpe1_315092
Linpe1_309503
Coere1_47663
Linpe1_220728
Ophsi1_EQK98796
Rhimi11_294234
RhimiATCC52814_232914
Rhich1_13828
RhimiATCC11559_227701
Rhich1_6824
Rhior3_7895
Mucci2_40585
Mucci2_76555
Mucci2_130546
RhimiATCC52814_300404
Rhimi11_250367
Rhich1_5243
RhimiATCC11559_149043
Rhich1_7776
Mucci2_134686
Rhior3_12046
Phybl2_101903
RhimiATCC52814_209200
Rhimi11_230814
Rhich1_9837
Rhich1_13261
Rhior3_8588
Rhior3_13744
Mucci2_165846
Phybl2_180075
Phybl2_118400
0.2

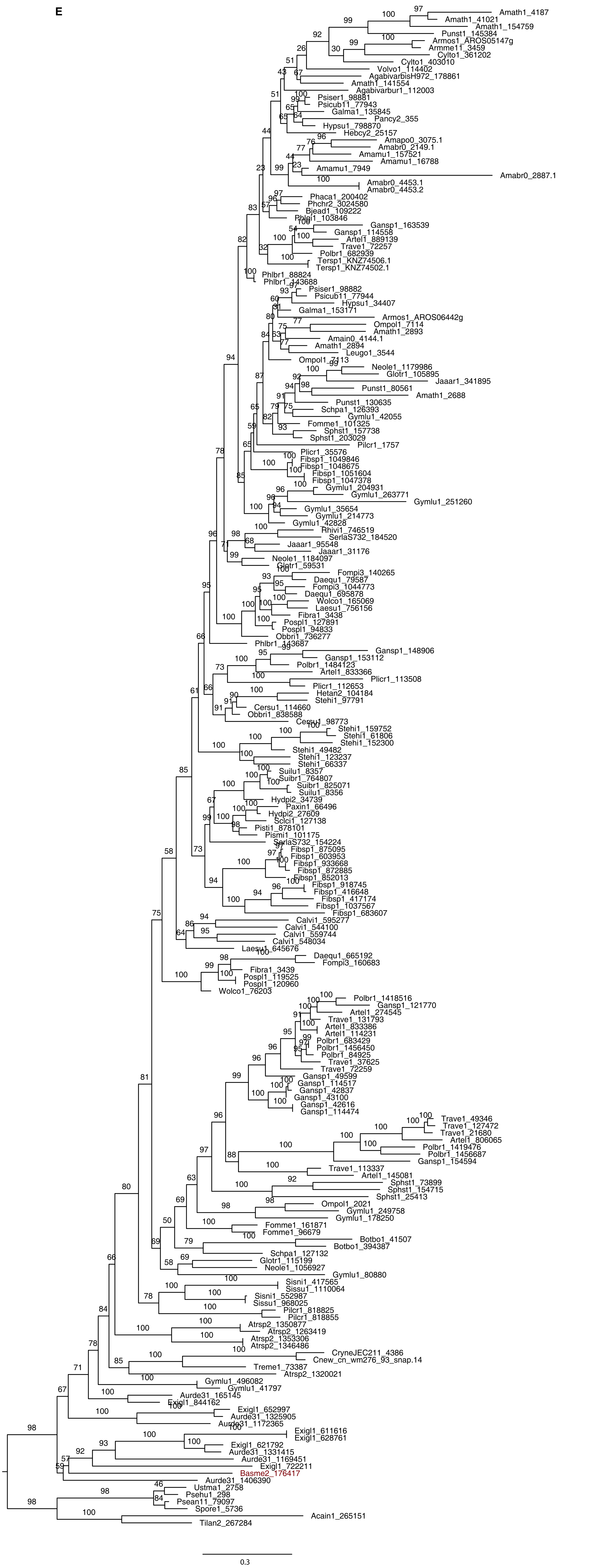
E
Amath1_4187
Amath1_41021
Amath1_154759
Punst1_145384
Armos1_AROS05147g
Armme11_3459
Cylto1_361202
Cylto1_403010
Volvo1_114402
AgabivarbisH972_178861
Amath1_141554
Agabivarbur1_112003
Psiser1_98881
Psicub11_77943
Galma1_135845
Pancy2_355
Hypsu1_798870
Hebcy2_25157
Amapo0_3075.1
Amabr0_2149.1
Amamu1_157521
Amamu1_16788
Amamu1_7949
Amabr0_2887.1
Amabr0_4453.1
Amabr0_4453.2
Phaca1_200402
Phchr2_3024580
Bjead1_109222
Phlgi1_103846
Gansp1_163539
Gansp1_114558
Artel1_889139
Trave1_72257
Polbr1_682939
Tersp1_KNZ74506.1
Tersp1_KNZ74502.1
Phlbr1_88824
Phlbr1_143688
Psiser1_98882
Psicub11_77944
Hypsu1_34407
Galma1_153171
Armos1_AROS06442g
Ompol1_7114
Amath1_2893
Amain0_4144.1
Amath1_2894
Leugo1_3544
Ompol1_7113
Neole1_1179986
Glotr1_105895
Jaaar1_341895
Punst1_80561
Amath1_2688
Punst1_130635
Schpa1_126393
Gymlu1_42055
Fomme1_101325
Sphst1_157738
Sphst1_203029
Pilcr1_1757
Plicr1_35576
Fibsp1_1049846
Fibsp1_1048675
Fibsp1_1051604
Fibsp1_1047378
Gymlu1_204931
Gymlu1_263771
Gymlu1_251260
Gymlu1_35654
Gymlu1_214773
Gymlu1_42828
Rhivi1_746519
SerlaS732_184520
Jaaar1_95548
Jaaar1_31176
Neole1_1184097
Glotr1_59531
Fompi3_140265
Daequ1_79587
Fompi3_1044773
Daequ1_695878
Wolco1_165069
Laesu1_756156
Fibra1_3438
Pospl1_127891
Pospl1_94833
Obbri1_736277
Phlbr1_143687
Gansp1_148906
Gansp1_153112
Polbr1_1484123
Artel1_833366
Plicr1_113508
Plicr1_112653
Hetan2_104184
Stehi1_97791
Cersu1_114660
Obbri1_838588
Cersu1_98773
Stehi1_159752
Stehi1_61806
Stehi1_152300
Stehi1_49482
Stehi1_123237
Stehi1_66337
Suilu1_8357
Suibr1_764807
Suibr1_825071
Suilu1_8356
Hydpi2_34739
Paxin1_66496
Hydpi2_27609
Sclci1_127138
Pisti1_878101
Pismi1_101175
SerlaS732_154224
Fibsp1_875095
Fibsp1_603953
Fibsp1_933668
Fibsp1_872885
Fibsp1_852013
Fibsp1_918745
Fibsp1_416648
Fibsp1_417174
Fibsp1_1037567
Fibsp1_683607
Calvi1_595277
Calvi1_544100
Calvi1_559744
Calvi1_548034
Laesu1_645676
Daequ1_665192
Fompi3_160683
Fibra1_3439
Pospl1_119525
Pospl1_120960
Wolco1_76203
Polbr1_1418516
Gansp1_121770
Artel1_274545
Trave1_131793
Artel1_833386
Artel1_114231
Polbr1_683429
Polbr1_1456450
Polbr1_84925
Trave1_37625
Trave1_72259
Gansp1_49599
Gansp1_114517
Gansp1_42837
Gansp1_43100
Gansp1_42616
Gansp1_114474
Trave1_49346
Trave1_127472
Trave1_21680
Artel1_806065
Polbr1_1419476
Polbr1_1456687
Gansp1_154594
Trave1_113337
Artel1_145081
Sphst1_73899
Sphst1_154715
Sphst1_25413
Ompol1_2021
Gymlu1_249758
Gymlu1_178250
Fomme1_161871
Fomme1_96679
Botbo1_41507
Botbo1_394387
Schpa1_127132
Glotr1_115199
Neole1_1056927
Gymlu1_80880
Sisni1_417565
Sissu1_1110064
Sisni1_552987
Sissu1_968025
Pilcr1_818825
Pilcr1_818855
Atrsp2_1350877
Atrsp2_1263419
Atrsp2_1353306
Atrsp2_1346486
CryneJEC211_4386
Cnew_cn_wm276_93_snap.14
Treme1_73387
Atrsp2_1320021
Gymlu1_496082
Gymlu1_41797
Aurde31_165145
Exigl1_844162
Exigl1_652997
Aurde31_1325905
Aurde31_1172365
Exigl1_611616
Exigl1_628761
Exigl1_621792
Aurde31_1331415
Aurde31_1169451
Exigl1_722211
Basme2_176417
Aurde31_1406390
Ustma1_2758
Psehu1_298
Psean11_79097
Spore1_5736
Acain1_265151
Tilan2_267284
0.3

F

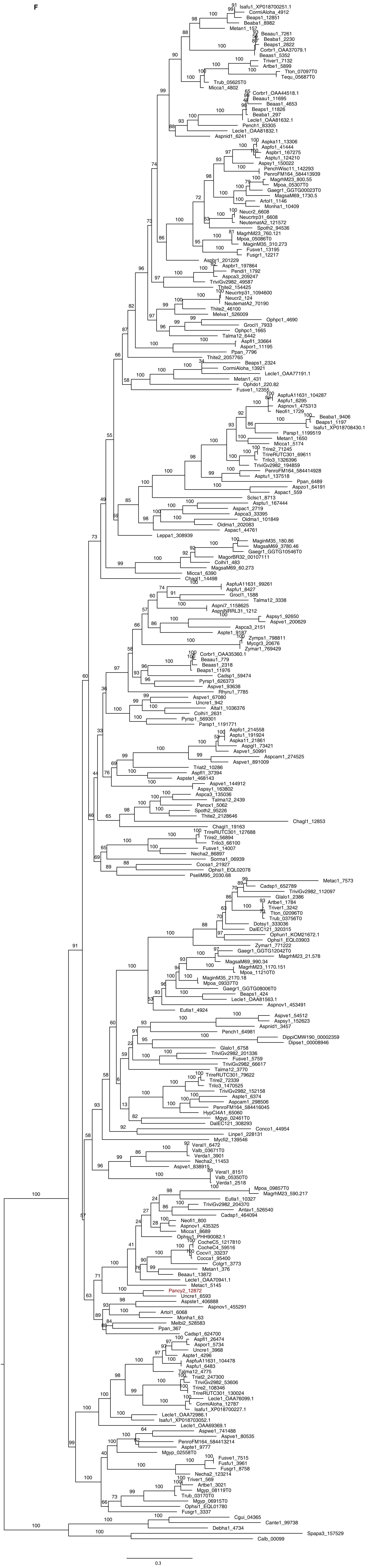

Supplemental Figure 3. Phylogeny of LysM domains

Maximum Likelihood phylogeny (IQ-tree) of LysM domains extracted from all fungal chitinases. Support values indicate percentage of rapid bootstraps

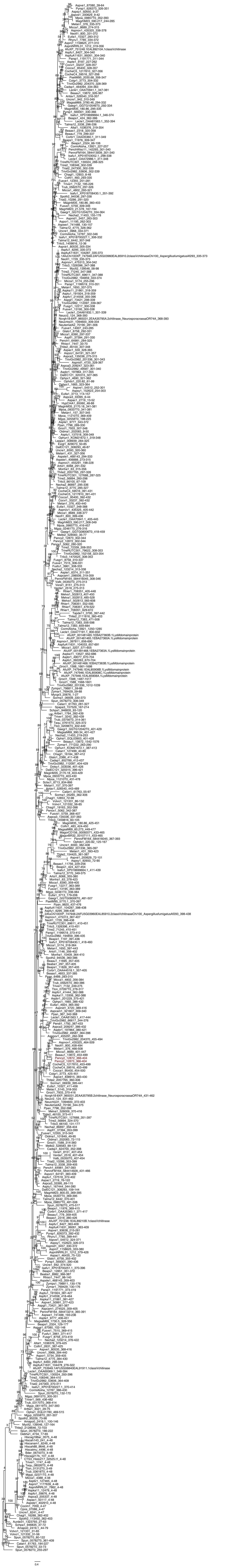

Supplement: msaa293_Supplementary_Data [file msaa293_supplementary_data.zip › Supplemental_Figures.pdf]
